# Incorporating the Patient Voice in Sarcoma Research: How Can We Assess Health-Related Quality of Life in This Heterogeneous Group of Patients? A Study Protocol

**DOI:** 10.3390/cancers13010001

**Published:** 2020-12-22

**Authors:** Dide den Hollander, Marco Fiore, Javier Martin-Broto, Bernd Kasper, Antonio Casado Herraez, Dagmara Kulis, Ioanna Nixon, Samantha C. Sodergren, Martin Eichler, Winan J. van Houdt, Ingrid M. E. Desar, Isabelle Ray-Coquard, Claire Piccinin, Hanna Kosela-Paterczyk, Aisha Miah, Leopold Hentschel, Susanne Singer, Roger Wilson, Winette T. A. van der Graaf, Olga Husson

**Affiliations:** 1 Department of Medical Oncology, Netherlands Cancer Institute, 1066 CX Amsterdam, The Netherlands; d.d.hollander@nki.nl (D.d.H.); w.vd.graaf@nki.nl (W.T.A.v.d.G.); 2 Department of Medical Oncology, Radboud University Medical Centre, 6525 GA Nijmegen, The Netherlands; ingrid.desar@radboudumc.nl; 3 Department of Surgery, Fondazione IRCCS Istituto Nazionale dei Tumori, 20133 Milan, Italy; Marco.Fiore@istitutotumori.mi.it; 4 Department of Medical Oncology, Virgen del Rocío University Hospital, 41013 Sevilla, Spain; jmartin@mustbesevilla.org; 5 Sarcoma Unit, University of Heidelberg, Mannheim University Medical Center, 68167 Mannheim, Germany; bernd.kasper@medma.uni-heidelberg.de; 6 Department of Medical Oncology, Hospital Clínico San Carlos, 28040 Madrid, Spain; antonio.casado@salud.madrid.org; 7 Quality of Life Department, European Organisation for Research and Treatment of Cancer, 1200 Brussels, Belgium; dagmara.kulis@eortc.org (D.K.); claire.piccinin@eortc.be (C.P.); 8 Department of Clinical Oncology, The Beatson Cancer Center, Glasgow G12 0YN, UK; ioanna.nixon@ggc.scot.nhs.uk; 9 School of Health Sciences, University of Southampton, Southampton SO17 1BJ, UK; S.C.Sodergren@soton.ac.uk; 10 University Cancer Center (NCT/UCC), University Hospital Carl Gustav Carus, Technical University Dresden, 01069 Dresden, Germany; martin.eichler@uniklinikum-dresden.de (M.E.); Leopold.Hentschel@uniklinikum-dresden.de (L.H.); 11 Department of Surgical Oncology, Netherlands Cancer Institute, 1066 CX Amsterdam, The Netherlands; w.v.houdt@nki.nl; 12 HESPER Lab, Department of Medical Oncology, Centre Léon-Bérard, University Claude Bernard Lyon, 69008 Lyon, France; isabelle.ray-coquard@lyon.unicancer.fr; 13 Department of Medical Oncology, Maria Sklodowska-Curie Memorial Cancer Center and Institute of Oncology, 02-034 Warsaw, Poland; Hanna.Kosela-Paterczyk@pib-nio.pl; 14 Department of Clinical Oncology, Royal Marsden NHS Foundation Trust, London SW3 6JJ, UK; aisha.miah@rmh.nhs.uk; 15 Epidemiology and Informatics, Institute of Medical Biostatistics, University Medical Centre Mainz, 55131 Mainz, Germany; singers@uni-mainz.de; 16 University Cancer Centre Mainz (UCT), University Medical Centre Mainz, 55131 Mainz, Germany; 17 Sarcoma Patients EuroNet, D-85521 Riemerling, Germany; roger.wilson@sarcoma-patients.eu; 18 Department of Medical Oncology, ErasmusMC Cancer Institute, Erasmus University Medical Center, 3015 GD Rotterdam, The Netherlands; 19 Division of Clinical Studies, Institute of Cancer Research, London SM2 5NG, UK

**Keywords:** sarcomas, soft tissue sarcoma, bone sarcoma, health-related quality of life, patient-reported outcomes

## Abstract

**Simple Summary:**

Sarcomas are a rare group of heterogeneous tumors. Because treatment side-effects detract from the survival benefit of treatment, it is important to assess treatment effectiveness in terms of patient-reported health-related quality of life (HRQoL). However, a sarcoma specific measurement instrument or strategy does currently not exist. This study aims to (1) develop a list of all HRQoL issues relevant to sarcoma patients; (2) determine a strategy for sarcoma-specific HRQoL assessment. An international, multicenter study will be conducted, searching existing literature and conducting interviews with adult sarcoma patients and healthcare professionals (HCPs) to create a list of HRQoL issues. Subsequently, another group of sarcoma patients and HCPs will be asked to rate and prioritize the issues. This information will help create a strategy to measure HRQoL in sarcoma patients, taking into account the heterogeneity of sarcomas, which will improve the collection of personalized patient-reported outcome data in future research and clinical care.

**Abstract:**

Sarcomas comprise 1% of adult tumors and are very heterogeneous. Long-lasting and cumulative treatment side-effects detract from the (progression-free) survival benefit of treatment. Therefore, it is important to assess treatment effectiveness in terms of patient-reported outcomes (PROs), including health-related quality of life (HRQoL) as well. However, questionnaires capturing the unique issues of sarcoma patients are currently lacking. Given the heterogeneity of the disease, the development of such an instrument may be challenging. The study aims to (1) develop an exhaustive list of all HRQoL issues relevant to sarcoma patients and determine content validity; (2) determine a strategy for HRQoL measurement in sarcoma patients. We will conduct an international, multicenter, mixed-methods study (registered at clinicaltrials.gov: NCT04071704) among bone or soft tissue sarcoma patients ≥18 years, using EORTC Quality of Life Group questionnaire development guidelines. First, an exhaustive list of HRQoL issues will be generated, derived from literature and patient (*n* = 154) and healthcare professional (HCP) interviews (*n* = 30). Subsequently, another group of sarcoma patients (*n* = 475) and HCPs (*n* = 30) will be asked to rate and prioritize the issues. Responses will be analyzed by priority, prevalence and range of responses for each item. The outcome will be a framework for tailored HRQoL measurement in sarcoma patients, taking into account sociodemographic and clinical variables.

## 1. Introduction

Sarcomas are a group of rare malignant solid neoplasms of mesenchymal origin, comprising more than 100 histological and more than 250 molecular subtypes [1]. Significant heterogeneity exists among cases with widely different patterns of the stage at diagnosis, tumor location at almost any anatomical site, prognosis, treatments, and age at diagnosis [2]. Sarcomas can be classified into soft tissue sarcomas (STS), including gastrointestinal stromal tumors (GIST) (84% of all sarcomas) and bone sarcomas (BS; 14%), and other sarcomas [3]. The overall incidence of sarcoma is about 7 in 100,000 persons, with 30,000 new cases a year in Europe [3].

Approximately 10% of patients present with de novo metastatic disease. Furthermore, many sarcomas have aggressive biological behavior, and around half of patients with high-grade tumors will eventually develop incurable metastatic disease [4,5]. The five-year relative survival in Europe (period 2000–2007) is 60% for STS and 50% for BS [6]; however, survival in the metastatic setting is poor; for example, in STS, median overall survival is 12–20 months [6,7] but may be many years in exceptional cases.

Treatment for localized STS usually consists of conservative surgery with pre- or postoperative radiotherapy, however in retroperitoneal STS extended multivisceral resection is often needed in order to obtain complete resection and reduce the risk of recurrence [8]. The evidence for (neo-)adjuvant chemotherapy is controversial; however, it may be considered in high-risk tumors. In patients with advanced STS, not amenable to curative surgical resection, systemic treatment with chemotherapy is the mainstay of therapy with the aim to relieve symptoms, slow tumor progression and prolong survival. In some sarcoma subtypes, other systemic options are available, including tyrosine kinase inhibitors [9,10,11], but access may be limited in some countries. Immunotherapy has shown some promise in trials of some advanced STS. However, no treatment has yet been approved [12]. Surgical resection, stereotactic ablative radiotherapy or local treatments such as radiofrequency ablation of oligo-metastases can be considered in selected cases [13]. Generally, conventional radiotherapy is applied in the palliative setting, mainly for pain reduction.

The treatment of BS also differs per subtype. In patients with localized chondrosarcoma, surgery alone is the standard of treatment. Ewing sarcoma and osteosarcoma patients will have surgery in addition to (neo)adjuvant chemotherapy. For Ewing sarcoma patients, radiotherapy can be delivered either instead of surgery, in cases where surgery would be morbid or significantly impact functional outcomes, but more often in the (neo) adjuvant setting. For patients with advanced BS, palliative chemotherapy and/or radiotherapy are potential therapeutic options.

Despite the effectiveness of treatments for sarcoma, the varying degrees of long-lasting and cumulative treatment side-effects and morbidity in a substantial number of patients contribute negatively to their overall outcome [8,14,15]. Information on survival only is insufficient to determine the net clinical (or true) benefit of a treatment for patients, and subjective patient-reported outcomes (PROs), including health-related quality of life (HRQoL), should be considered as well. HRQoL is a multidimensional concept that includes the patient’s perception of the impact of their disease and treatment on physical, psychological, and social functioning in daily life [16]. Measurement of HRQoL facilitates the calculation of quality-adjusted life years (QALY), defined as “the measure of the state of health of a person or group in which the benefits, in terms of length of life, are adjusted to reflect the quality of life” [17]. PROs are increasingly used as important endpoints in cancer clinical trials, and implementation has become more standardized [18,19]. Furthermore, studies have shown that using PROs in clinical practice leads to better patient-provider communication, more symptom control and patients are more willing to self-report their symptoms from home [20,21,22,23].

The availability of high-quality HRQoL data of sarcoma patients is limited but shows they have more physical and psychological problems than the general population [24,25]. Patients experience disabling disease- and treatment-related symptoms such as fatigue and pain, interference in family, social and vocational life, and limitations in leisure activities, which can, in turn, impact their mental health and confidence [14,26,27]. Existing HRQoL data in sarcoma patients is mostly based on the use of generic (SF-36, EQ-5D) or cancer-generic (EORTC-QLQ-C30, FACT-G) questionnaires; however, these measures do not cover all sarcoma- or sarcoma treatment-specific problems. For example, in the PALETTE trial (EORTC 62072), a double-blind, randomized, phase 3 trial of pazopanib versus placebo as second-line or later treatment for patients with advanced STS, HRQoL was an exploratory endpoint, using the EORTC QLQ-C30 [28]. Although HRQoL did not improve with pazopanib, the observed improvement in progression-free survival without impairment of HRQoL was considered a relevant result. The toxicity profile of pazopanib was reflected in the patients’ self-reported symptoms (e.g., diarrhea) but did not translate into significantly worse overall global health status during treatment. However, several other side-effects common to angiogenesis inhibitors (e.g., hand-foot syndrome or skin reactions) were not assessed as PROs in this study. Another study reported that bone sarcoma survivors struggle with their altered appearance and display of functional impairments (e.g., walking with a limp or needing a crutch), which impacts their self-esteem and social life [29]. This is not covered by existing (cancer-)generic questionnaires. Thus, to fully understand and assess in detail the impact of this sarcoma treatment on all domains of HRQoL, in both clinical research and care, the use of HRQoL measure(s) that includes sarcoma-specific treatment symptoms or issues is needed.

Usually, the development of a tumor-specific module resolves the problem of insufficient content coverage of a generic measure. A comprehensive sarcoma-specific HRQoL measure for use in clinical trials is currently lacking, as existing HRQoL measures for sarcoma patients are focused on physical functioning only (e.g., Toronto extremity salvage score (TESS) as a measure of physical disability [30]) or patient experiences (e.g., sarcoma assessment measure (SAM) [31]). However, it was found during the development process of the SAM that tumor location and type of treatment received are important factors that determine which HRQoL issues subgroups of sarcoma patients are dealing with, in addition to established factors including sex and age [32,33]. For example, five issues (not specified) were identified to be only relevant in patients who had undergone amputation, and nine issues were relevant to head and neck sarcoma patients [31]. This heterogeneity of the disease was also reflected in HRQoL outcomes in German sarcoma patients and survivors [34]. These data show that the development of a single instrument is challenging, and it may not be the best option to meet the needs of academia and industry to assess the impact of new treatments [15,35].

The European Organization for Research and Treatment of Cancer Quality of Life Group (EORTC QLG) is currently developing a new measurement strategy, shifting to a more flexible approach that allows for the use of customized measures, in addition to the “static” standardized questionnaires, where relevant [36]. Traditionally, questionnaires are developed during a 4-phase process which takes several years, i.e., (1) generation of HRQoL issues, (2) construction of an item list, (3) pretesting the provisional questions, (4) field-testing to determine reliability, validity and responsiveness [37]. The new approach entails the use of a combination of standardized HRQoL questionnaires (i.e., the EORTC Quality of Life Questionnaire Core 30 (EORTC QLQ-C30) and existing disease-, site, or population-specific modules to enable population comparison, and validated items from the so-called Item Library (an online platform comprised of more than 900 individual items from over 60 EORTC questionnaires) to cover the full range of issues relevant for the study population (depending on the research question) [38]. Item banks also enable computer adaptive testing (CAT) techniques, tailoring the selection of items based on responses to previous items [39,40]. This flexible approach offers the possibility to encompass the full range of age-, location- and treatment-specific issues relevant to sarcoma patients and to improve the relevance for patients in answering questions that are meaningful to them. Furthermore, it also allows for shorter questionnaires, which makes compliance to a repeat routine more likely and opens the way to longitudinal measurement, which patients find improves understanding [41].

This study aims to develop an exhaustive list of all HRQoL issues relevant to sarcoma patients (irrespective of their sarcoma stage, subtype, treatment, age) and to determine content validity. Subsequently, a strategy for HRQoL measurement in sarcoma patients will be determined that will allow for tailored, longitudinal assessment based on sociodemographic and clinical variables in future clinical trials.

## 2. Methods and Analysis

### 2.1. Study Design and Setting

We will conduct a cross-sectional, international, multicenter, mixed-method phase 1 questionnaire development study among bone and soft tissue sarcoma patients at more than thirty centers world-wide, following EORTC QLG questionnaire development guidelines [37]. First, a list of HRQOL issues will be generated, derived from the available literature and patient- and healthcare professional (HCP) interviews. Subsequently, another group of sarcoma patients and HCPs will be asked to rate the issues on relevance and to prioritize the most important issues. Clinical data will be extracted from patient records. The study started already with recruitment at the first center in May 2019, and a systematic review of the literature has recently been published [42].

### 2.2. Patient and Public Involvement

Implementation of HRQoL and other PRO assessments in sarcoma research is one of the research priorities of the Sarcoma Patients EuroNet (SPAEN), an international network of sarcoma, GIST, and Desmoid patient advocacy groups. Furthermore, public involvement is an integral aspect of patient-reported outcome measure (PROM) development [43]. Patient representatives were involved in the design of the study (feedback on the protocol and patient information form) and will be involved in reporting the results. In several countries, local patient advocacy groups will disseminate information about this study among their members to increase awareness about this project and to encourage participation. Study results will also be shared with patients through these organizations.

### 2.3. Eligibility Criteria

Inclusion criteria are: (1) confirmed diagnosis of sarcoma (according to the ICD-10 codes C40 and C41 for bone sarcoma and C49 for soft-tissue sarcoma). Patients with all stages of the disease, and both during treatment or posttreatment follow-up, will be included. The primary types of treatment to be included are surgery, radiotherapy, systemic treatment, or a combination; (2) age ≥ 18 years. Although sarcoma is a disease affecting all ages and sarcoma patients aged < 18 years are seen within adult health services in some countries, only those aged 18 years and older will be included because the EORTC QLQ-C30 is only validated among cancer patients aged 18 years and older. The EORTC Quality of Life Group is currently conducting a study to identify unique HRQoL issues specific to adolescents and young adults (AYAs; aged 14–39 years) with cancer and to validate the EORTC QLQ-C30 in young people with cancer [44]. Depending on the results of this study, a measurement strategy for AYA sarcoma patients can be determined at a later stage (3) having the mental capacity to provide informed consent and participate in the study (as determined by their treating physician). Patients must be able to rate or complete questionnaires themselves, as a prerequisite for patient-reported outcome measures; (4) medical professionals, e.g., surgical oncologist, medical oncologist, radiation oncologist or clinical oncologist, nurse, social worker, psychologist, a physiotherapist with experience of working with sarcoma patients.

Exclusion criteria are: (1) being too ill (on the judgment of the treating physician); the following diagnoses: (2) GIST; (3) Kaposi sarcoma; (4) carcinosarcoma; (5) benign and locally aggressive mesenchymal tumors.

### 2.4. Data Collection

#### 2.4.1. Literature Review

First, a search of the literature through four medical search engines has been performed to identify all relevant HRQoL issues and existing HRQoL questionnaires currently used among sarcoma patients. The search strategy was created with the help of a librarian and combined medical subject headings (MeSH) terms and keywords for sarcoma and quality of life [42]. The reference lists of all identified publications will be reviewed to retrieve other relevant publications. There will be no restrictions with regard to the year of publication. This literature search will result in a list of HRQoL issues relevant to sarcoma patients, which will be used to develop the issue list.

#### 2.4.2. Part A: Interviews

First, the respondent will be asked an open question about his/her sarcoma history and experience and then be prompted according to the interview schedules (one for patients and one for HCPs) that will be provided for all collaborators to ensure homogeneity in data collection (Supplementary Material, text S1). Subsequently, the respondent will be asked to review the EORTC QLQ-C30 and a site-specific module, if available, for the topic and item relevance (to be scored on a 4-point Likert scale of relevance for the survivorship period). Those with a sarcoma in the head and neck region will be asked to complete the EORTC QLQ-H&N43 and those with a uterine sarcoma the EORTC QLQ-EN24 to determine the relevance of these existing site-specific items of these questionnaires in sarcoma patients. All patients will receive a copy in their own language. The respondent will be encouraged to “think aloud”, providing feedback on the reasons for his/her ratings. The respondents will also be asked to identify HRQoL issues that they believe to be important that are not included in the current core questionnaire and, where relevant, site-specific module.

This list of HRQoL issues generated by the literature search and semi-structured patient and HCP interviews will be consolidated into a comprehensive list of issues for all languages of collaborating countries.

#### 2.4.3. Part B: Review Issue-List

The new list of HRQoL issues will be administered to another group of sarcoma patients and HCPs. Patients and HCPs will be asked to rate the HRQoL issues on relevance (4-point Likert scale: (1) not relevant–(4) very relevant), to prioritize the 25 most important issues, and to indicate relevant issues missing from this list. Patients will also be asked to complete the EORTC QLQ-C30. In addition, those with a sarcoma in the head and neck region are asked to complete the EORTC QLQ-HN43 and those with a uterine sarcoma the EORTC QLQ-EN24.

### 2.5. Recruitment

Patients will be recruited from Northern Europe (e.g., Germany, The Netherlands, Norway), Central/Eastern Europe (e.g., Poland), Southern Europe (e.g., Cyprus, France, Italy, Spain), the United Kingdom and also in countries outside Europe, including Australia, Canada, Hong Kong, India, Israel, Jordan, and the United States.

The study coordinator will use stratification to ensure that subjects represent the target population and will communicate to the local collaborators which patients can be invited. Purposive sampling will aim to achieve good representation according to sex, age, sarcoma location and treatment. When selecting the patients for interviews, care will be taken to include both younger and older patients, males and females, all types of treatment, as well as newly diagnosed patients and patients in posttreatment follow-up. We will stratify our sample according to the 6 locations of sarcoma (upper and lower extremities, axial skeleton, retroperitoneal/intra-abdominal, head and neck including the scalp, thorax including breast, and pelvic organs including urogenital), disease stage (localized, metastatic disease/local relapse) and subtype (bone sarcoma, soft tissue sarcoma) to ensure the heterogeneity of sarcomas in terms of subtypes, treatments, age, sex, and physical impairments is adequately covered (Table 1). These characteristics are based on the clinical experience of health care professionals and researchers in the sarcoma field and input from patient representatives.

During part A, eligible patients will be identified from the Medical Oncology, Radiation Oncology or Surgery departments of the participating hospitals and through patient organizations. The health care professional will introduce the study to eligible patients. If a patient expresses interest, he or she will be approached by the researcher for further details about the study, the interview, and the agreement to participate.

In part B, patients will be selected by the clinical team in the participating hospitals and invited to participate in the study using an invitation letter signed by the treating physician or in person during a routine follow-up appointment. Within one week, they will be contacted by phone to explain the study and to ask if they are willing to participate. If they are willing to participate, they will receive by mail an informed consent form, the issue list and the EORTC QLQ-C30 questionnaire (and EORTC QLQ-H&N43 or -EN24 module if applicable).

During both part A and part B, HCPs with experience of working with sarcoma patients will be invited by the study coordinator or the local PI.

### 2.6. Case Report Forms

Sociodemographic data will be collected at study entry, including age, gender, level of education and employment status, living arrangements, travel distance to hospital, and access to centralized care. These data may be extracted from the dossier or obtained from the patient directly. Clinical data will also be collected, including primary diagnosis, stage of the disease, type of therapy (if any), and comorbidity using the Charlson comorbidity index and international prognostic index (IPI) [45]. These clinical data will be extracted from the medical file and noted on the demographic and clinical data form. The information will be recorded and stored in accordance with the Dutch General Data Protection Regulation. Data linkage will be done at the Netherlands Cancer Institute.

### 2.7. Questionnaires

Three existing EORTC quality of life questionnaires will be reviewed by patients and HCPs, for the topic and item relevance. The EORTC QLQ-C30 is provided for all patients, the EORTC QLQ-H&N43 for patients with sarcoma in the head and neck region and the EORTC QLQ-EN24 for patients with uterine sarcoma. Before the start of phase 1b, based on the issues found in phase 1a, it will be evaluated if more location-specific modules (e.g., prostate, breast or colorectal) can be included. All scales and single-item measures range from 0 to 100 after linear transformation. A higher score on functional scales and global QoL means better functioning and HRQoL, whereas a higher score on the symptom scales means more complaints.

#### 2.7.1. EORTC-Quality of Life Questionnaire (EORTC QLQ-C30)

The EORTC QLQ-C30 version 3.0 was developed to measure HRQoL in patients with cancer [46]. It was translated into and linguistically validated into over 110 languages. This 30-item questionnaire consists of five functional scales (physical, role, cognitive, emotional and social), a global health status/quality of life scale, three symptom scales (fatigue, pain, nausea and vomiting) and six single items assessing common symptoms (dyspnea, loss of appetite, insomnia, constipation and diarrhea) and perceived financial impact of the disease.

#### 2.7.2. EORTC-QLQ-Head and Neck Module (EORTC QLQ-H&N43)

The EORTC QLQ-Head and Neck Module (EORTC QLQ-H&N43) measures HRQoL in patients with head and neck cancer [47]. It has been translated into and linguistically validated in 29 languages. It consists of 43 items, divided into 12 symptom scales (pain in the mouth, swallowing, problems with teeth, dry mouth and sticky saliva, problems with senses, speech, body image, social eating, sexuality, problems with shoulder, skin problems and fear of progression) and 7 single items (problems opening the mouth, coughing, social contact, swelling in the neck, weight loss, problems with wound healing and neurological problems).

#### 2.7.3. EORTC-QLQ-Endometrial Cancer Module (EORTC QLQ-EN24)

The EORTC QLQ-Endometrial Cancer Module (EORTC QLQ-EN24) was developed for HRQoL measurement in patients with endometrial cancer and consists of 24 items [48]. It has been translated into and linguistically validated in 35 languages. Five symptom scales (lymphedema, urological symptoms, gastrointestinal symptoms, poor body image and sexual/vaginal problems), five single symptoms items (pain in back and pelvis, tingling/numbness, muscular pain, hair loss and taste change) and three single functional items (sexual interest, sexual activity and sexual enjoyment) are measured.

### 2.8. Objectives

The primary objective is to develop an HRQoL measurement strategy for sarcoma patients, indicating which issues can be included for all sarcoma patients (sarcoma-generic) and which issues should be evaluated in certain subgroups (e.g., based on treatment or sarcoma location) only. This is the first step towards the implementation of sarcoma specific PROs in future clinical trials. Secondary endpoints are: (1)to develop an exhaustive list of all HRQoL issues relevant to sarcoma patients (irrespective of their sarcoma subtype, treatment, time since diagnosis and age, and including generic/specific issues), (2) to determine the proportion of patients in each subgroup (according to tumor localization, type of sarcoma, disease stage and treatment) rating each item and the items from the EORTC QLQ C30 as relevant (i.e., rating 2–4 on Likert scale indicates relevance), (3) to determine the proportion of respondents with head neck or uterine localization rating each site-specific item of the H&N43 or EN24 questionnaire as relevant (i.e., rating 2–4 on Likert scale indicates relevance), (4) to determine the proportion of subgroups rating each item as relevant (i.e., half subgroups rating an item as relevant indicates a sarcoma-generic issue), and (5) to determine subgroup-specific (according to localization, type of treatment, subtype and disease stage) issues using a comparative approach (issues cited at least 1.5 times more indicates subgroup-specificity).

### 2.9. Sample Size Calculation

Part A: No formal sample size calculation can be made for the interviews. Data collection continues until saturation is reached, which is usually after 12 patients in phenomenological research [49]. Saturation occurs when adding more participants to the study (or, in this case, subgroup) does not result in additional perspectives or information. A stratification matrix (Table 1) will be used that ensures that at least 12 patients per location and 10 per treatment will be interviewed, considering the incidence of sarcoma location and treatment. In total, 154 patients will be recruited. Although some locations will be overrepresented in this sample compared to the population-based incidence numbers, this proportion was chosen to make sure that enough patients are enrolled per group to be able to conduct subgroup analyses.

Part B: Although the EORTC guidelines for development state that no more than 10 patients should be included in this part, the heterogeneity of the sarcoma population requires greater numbers for adequate subanalyses. Again, at least 10 patients per location and 10 per treatment will be recruited (Table 1), leading to a total of 475 patients.

Healthcare professionals: According to the EORTC module development guidelines, at least 5 HCPs should be included; therefore, 5 HCPs will be interviewed for each location (i.e., 6 in total), which means 30 HCPs in total. This applies to both parts A and B.

### 2.10. Statistical Analysis

We will perform thematic analysis of the qualitative data. Transcripts of the patient and HCP interviews will be examined by two coders in detail in order to identify basic patterns and recurrent themes using line-by-line coding to examine, compare and begin to develop conceptual categories. Categories will be developed inductively using the constant comparison method. Comparing each item with the rest of the data to create analytical categories, and then grouping categories together will make it possible to identify key themes [50]. The consolidated criteria for reporting qualitative research (COREQ) will be followed to ensure the accuracy of this qualitative study [51].

Data from patient and HCP interviews and questionnaires will be analyzed according to the EORTC module development guidelines [36], using basic quantitative analyses, including descriptive statistics (e.g., missing data, means and standard deviations, floor and ceiling effects), prevalence (number of patients who experienced each complaint, i.e., who scored 2, 3 or 4, divided by the total number of patients who completed that item, multiplied by 100), priority ratings (number of patients and professionals who have given priority ratings to each item) and range of responses for each item.

In order to facilitate transparent decision-making concerning the best approach to take in developing a sarcoma measurement strategy, the existing decision criteria will be followed: (1) an inclusive approach will be taken with regard to relevant items; (2) inclusion of an issue will be considered when more than 30% of patients (in total or per subgroup) mention the issue as missing; (3) issues that have a low (e.g., mean <1.5) mean score for relevance or importance will be considered for exclusion.

To distinguish “sarcoma generic” from “subgroup-specific issues”, issues that are more or less frequent in specific subgroups will be evaluated. An issue will be coded as endorsed by a respondent if it was scored 2 (“a little relevant”) or higher on the 4-point response scale. An issue needs to be endorsed by at least 30% of the patients in a subgroup to be included as an issue in that subgroup. If an issue is endorsed by patients from 3 out of 6 tumor locations and 2 out of 4 treatment groups, it will be considered to be a generic sarcoma issue; otherwise, it can be considered a sarcoma subgroup-specific issue.

Additional analyses will be performed per subgroup for location, treatment(s) received (surgery; chemotherapy/targeted therapy; radiotherapy; no treatment), type of sarcoma (soft tissue vs. bone sarcoma), and disease stage (localized vs. metastatic). An issue needs to be endorsed by at least 30% of the patients in a subgroup, and the mean score needs to be 1.5 or higher to be included as an issue in a subgroup. Then the prevalence of each issue between the aforementioned subgroups (according to location, type of treatment, subtype and disease stage and combinations of these factors) will be compared to decide whether an issue is subgroup-specific. Issues that are cited by at least 1.5 times as many respondents in group A compared to group B (a ratio of 3:2) may be considered as subgroup-specific.

For head and neck, and uterine sarcoma patients, the scores on the H&N43 and EN24 will be analyzed. We will assess the prevalence (percentage of the subjects who score higher than 2 is >30% and a mean score of 1.5 or higher) of each site-specific item. When at least 80% of the items are prevalent, using the site-specific module for those sarcoma locations will be recommended.

All the collected information will help to advise researchers as to which factors need to be taken into account when creating an item list for a specific study focusing on subgroups of sarcoma patients.

### 2.11. Missing Data

We will describe the number of missing items (i.e., number of items or questions that were not rated for relevance by patients or HCPs) and describe these results in our findings.

## 3. Conclusions

Sarcoma diagnosis and treatment have a substantial impact on a patient’s HRQoL, which should be taken into account in research and organization of care for this patient group. This study will be the first to investigate the optimal strategy for the assessment of HRQoL in sarcoma patients. Measurement of HRQoL in the sarcoma population is challenging because of heterogeneity in terms of locations, subtypes, treatments, age, and prognosis, and a holistic measurement strategy or instrument does currently not exist. The multicenter, international design of the study enables adequate sample sizes for specific subgroups of this rare cancer and ensures a multi-ethnic background of participants. Furthermore, the use of purposive sampling allows for coverage of the previously described heterogeneity of sarcomas. Our results will help to ensure the collection of high-quality, personalized patient-reported outcome data in future clinical trials and observational studies, eventually improving tailored supportive care for sarcoma patients.

## Figures and Tables

**Table 1:** Stratification matrix phase 1a and 1b.

Stratification by Disease Stage, Subtype and Treatment	Extremities	Axial Skeleton (Including Pelvic Bones)	Head and Neck (Including Scalp)	Thoracic (Including Breast)	Retroperitoneal and Intra-Abdominal	Pelvic Organs Including Urogenital	Total
**Phase 1a**							
*Localized*							
Bone							
SU	4	2	2	- ^a^	-	-	
SU + RT	4	4	-				74
SU(+RT) + ST	4	4	-				
Soft tissue							
SU	2	-	2	6	6	10	
SU+RT	8	-	2	4	4	-	
*Metastatic/local relapse*							80
Bone							
Local treatment (+ST)	4	2	2	- ^a^	-	-	
ST	6	8	8		-	-	
Soft tissue							
Local treatment (+ST)	2	-	-	-	-	-	
ST	6	-		8	8	8	
BSC	2	-	2	2	2	2	10
**Total**							
Per location							154
Per treatment							
SU only:							34
SU + RT:							26
SU + RT + ST:							14
Surgery total:							74
RT total:	42	20	32	20	20	20	40
ST only:							60
ST total:							74
Local:							10
Per subtype							
Bone:							54
Soft tissue:							100
**Phase 1b**							
*Localized*							
Bone							
SU	20	4	5	- ^a^	-	-	
SU + RT	20	8	-				
SU(+RT) + ST	20	8	-				235
Soft tissue							
SU	10	-	4	12	24	20	
SU + RT	40	-	4	8	16	-	
SU(+RT) + ST	-	-	12	-	-	-	
*Metastatic/local relapse*							
Bone							
Local treatment (+ST)	20	4	4	- ^a^	-	-	
ST	30	16	16		-	-	240
Soft tissue							
Local treatment (+ST)	10	-	-	-	-	-	
ST	30	-	16	16	32	16	
BSC	10	-	4	4	8	4	30
**Total**							
Per location	210	40	65	40	80	40	475
Per treatment							
SU only:							99
SU + RT:							96
SU + RT + ST:							40
Surgery total:							235
RT total:							136
ST only:							172
ST total:							212
Local:							38
Per subtype							
Bone:							175
Soft tissue:							300

^a^ Covered by axial group; SU = surgery; ST = systemic therapy (e.g., chemotherapy, tyrosine kinase inhibitors, immunotherapy); RT = radiotherapy; BSC = best supportive care; local treatment = SU and/or RT in metastatic setting.

## Supplementary Materials

The following are available online at https://www.mdpi.com/2072-6694/13/1/1/s1, Text S1: Interview scripts for phase 1a (patients and HCPs).
